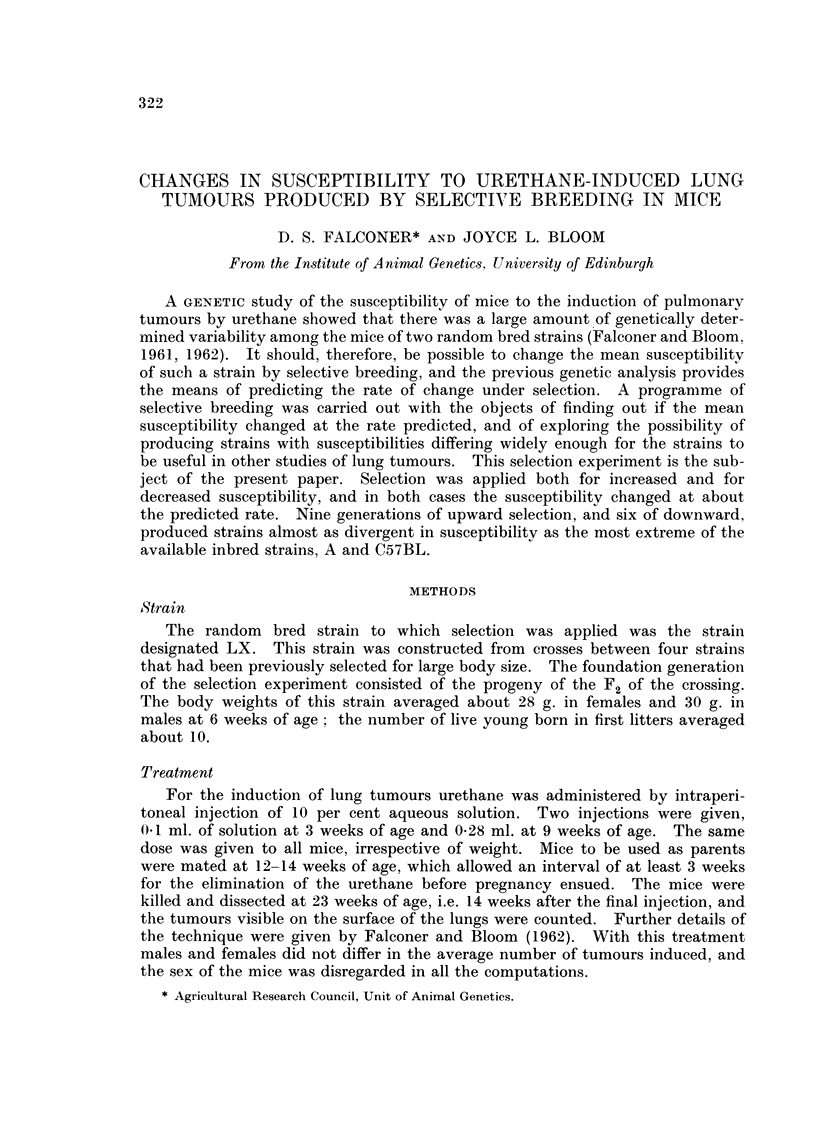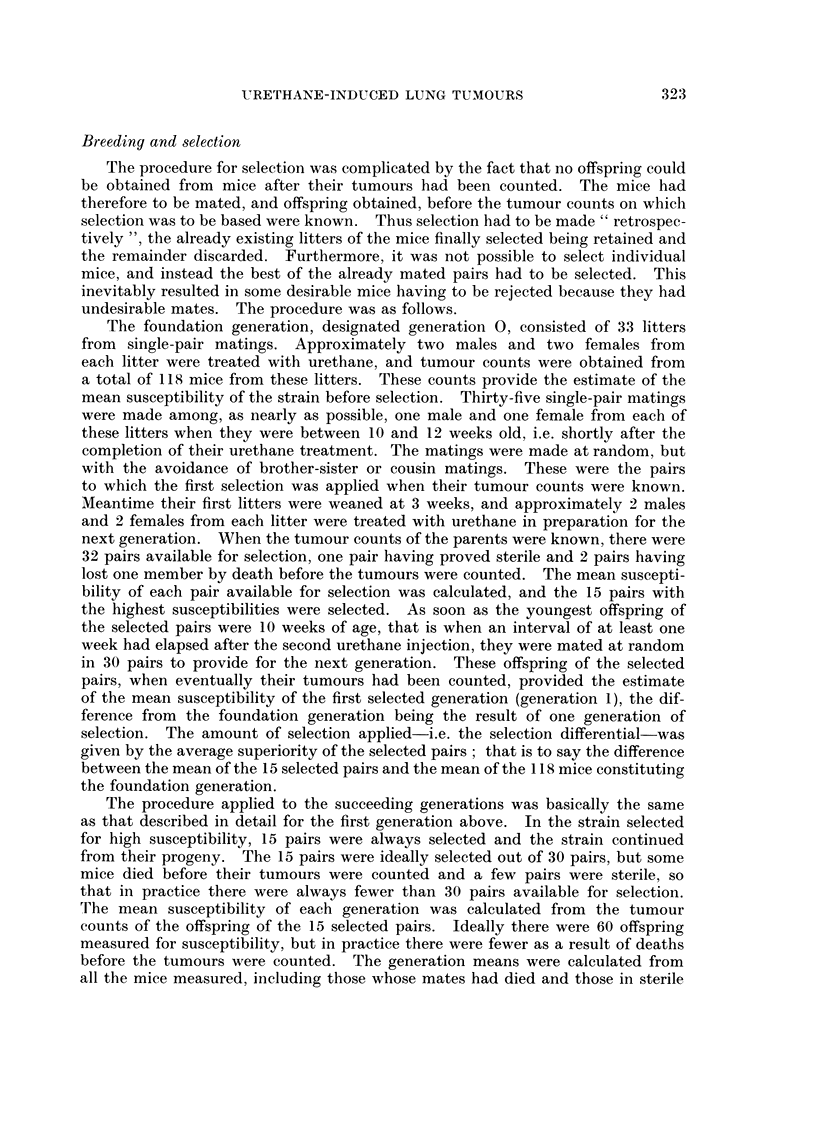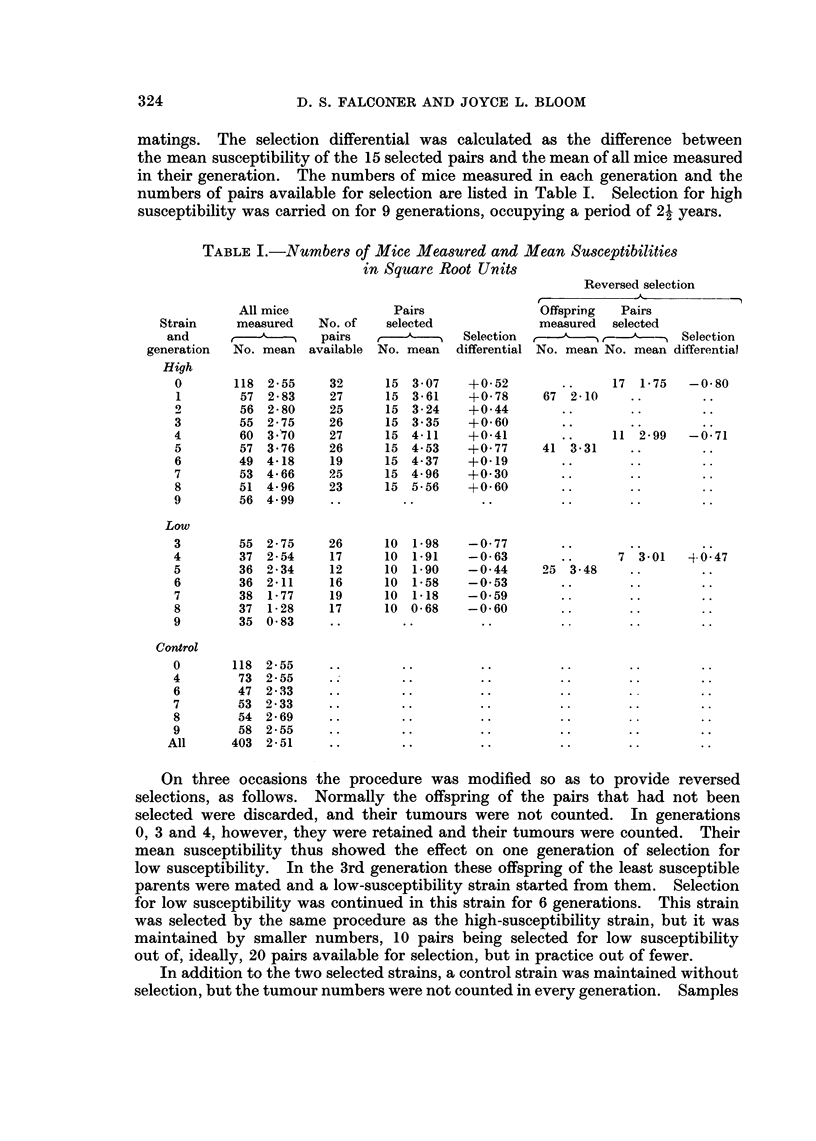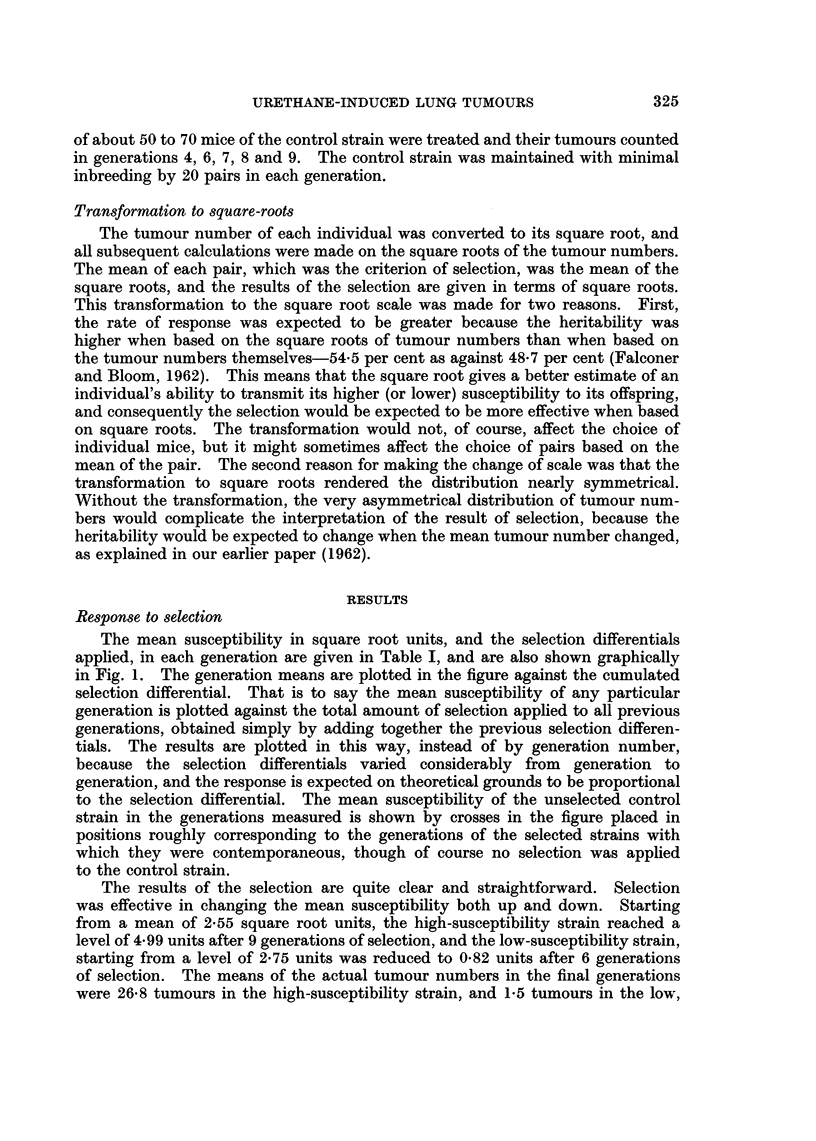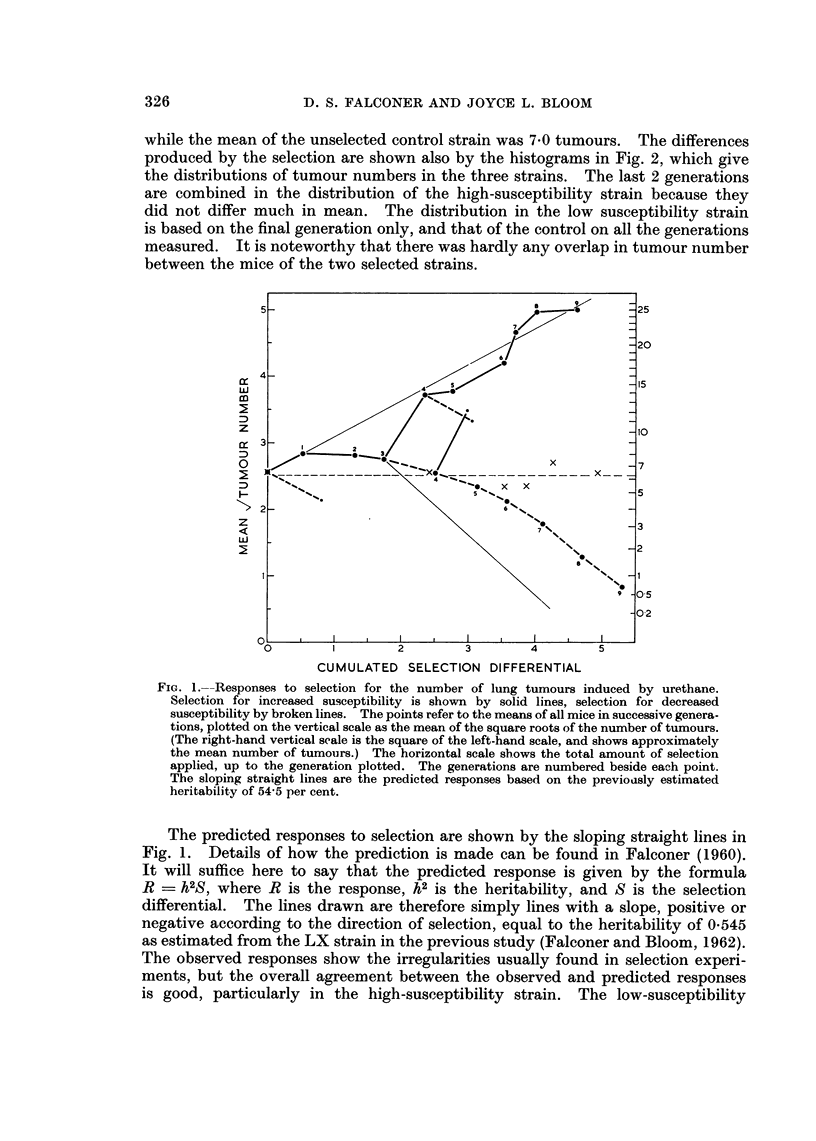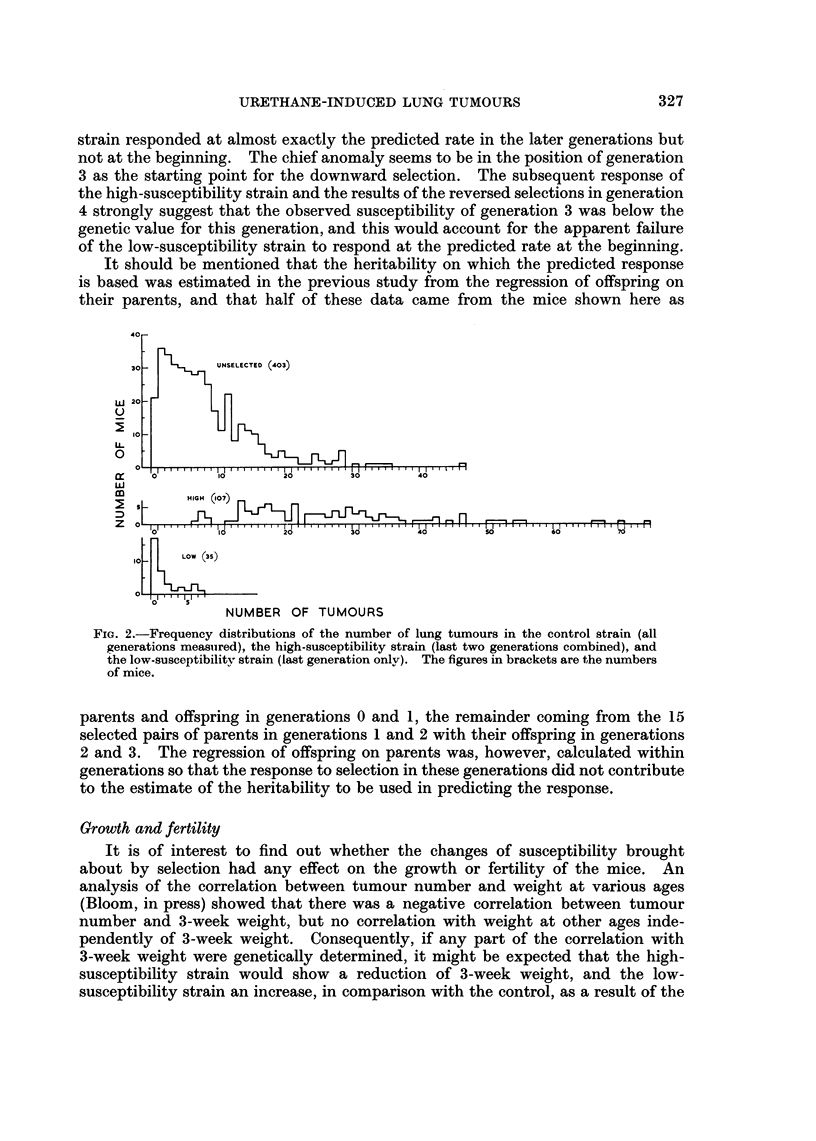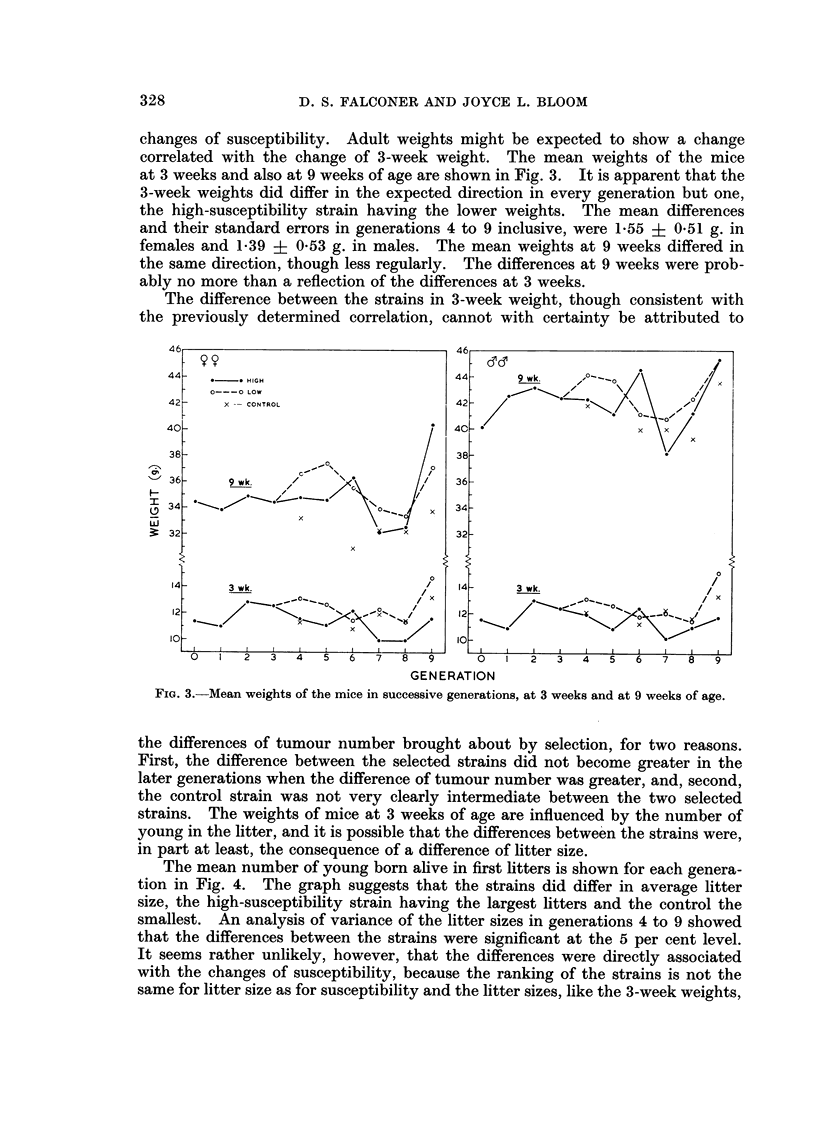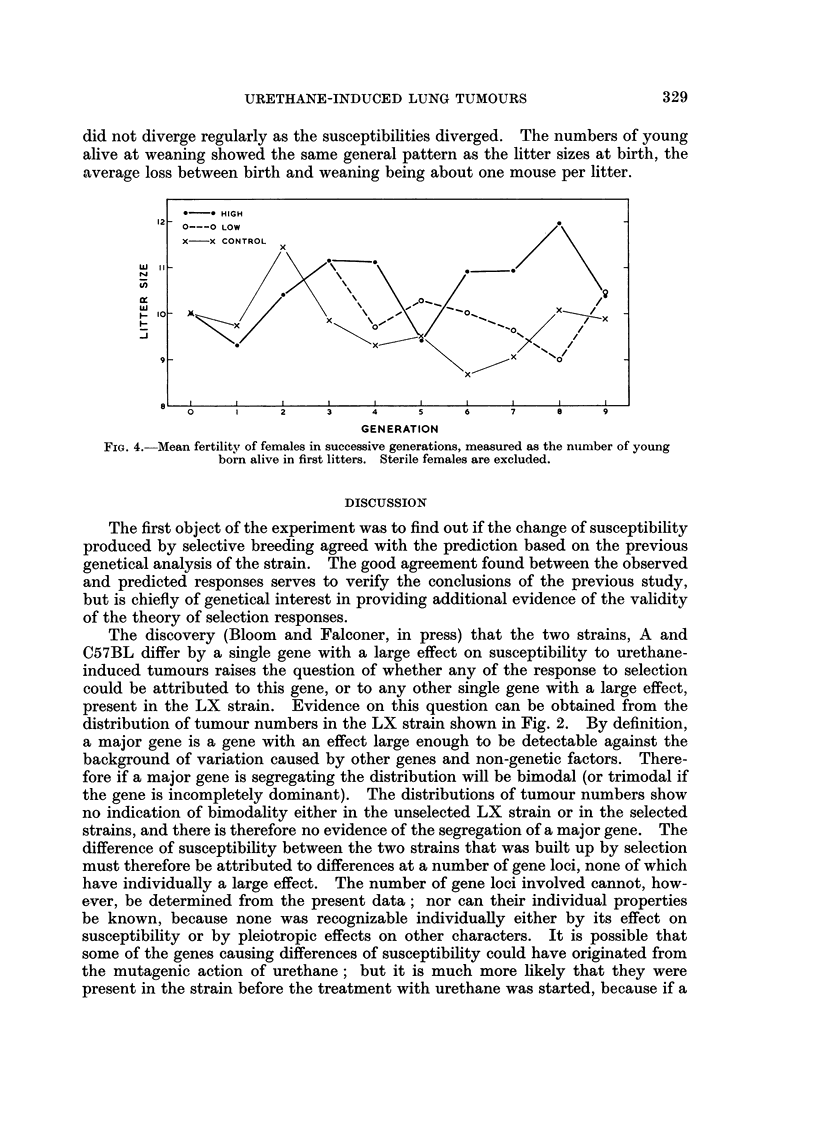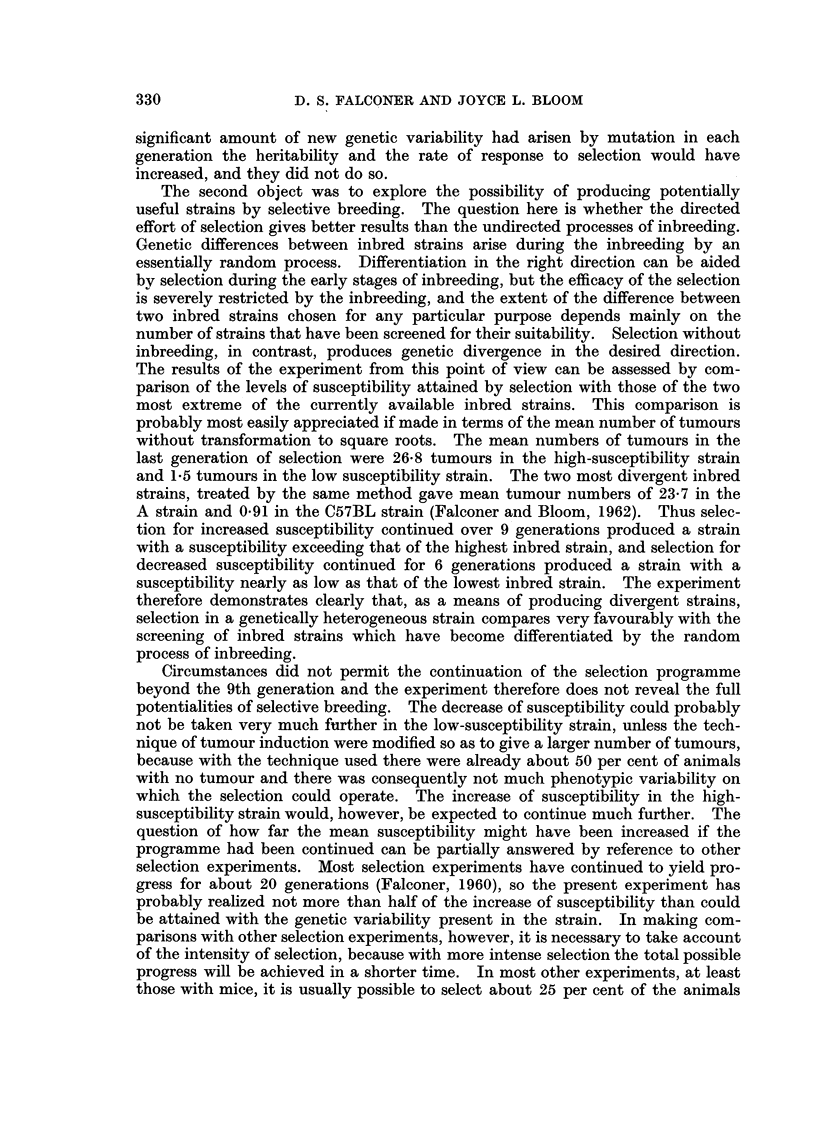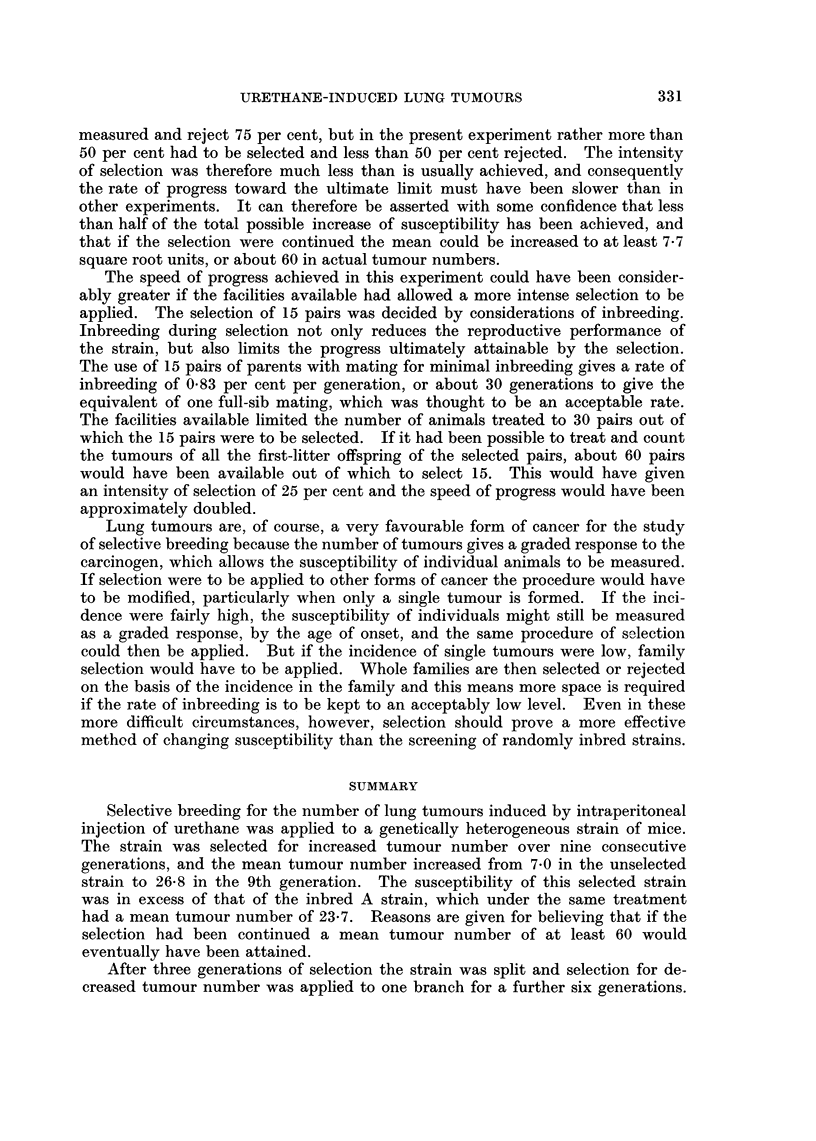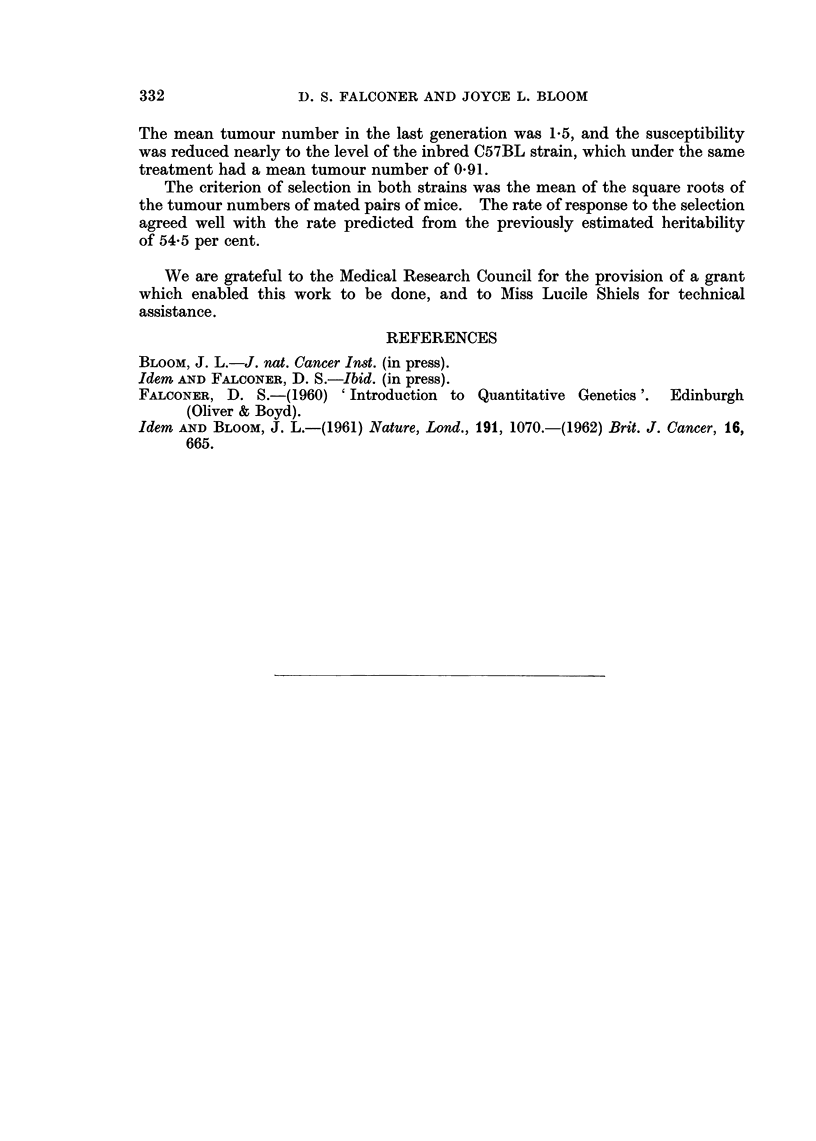# Changes in Susceptibility to Urethane-Induced Lung Tumours Produced by Selective Breeding in Mice

**DOI:** 10.1038/bjc.1964.38

**Published:** 1964-06

**Authors:** D. S. Falconer, Joyce L. Bloom


### CHANGES IN SUSCEPTIBILITY TO URETHANE-INDUCED LUNG TUMOURS PRODUCED BY SELECTIVE BREEDING IN MICE

D. S. FALCONER* AND JOYCE L. BLOOM

*From the Institute of Animal Genetics, University of Edinburgh*

A GENETIC study of the susceptibility of mice to the induction of pulmonary tumours by urethane showed that there was a large amount of genetically determined variability among the mice of two random bred strains (Falconer and Bloom, 1961, 1962). It should, therefore, be possible to change the mean susceptibility of such a strain by selective breeding, and the previous genetic analysis provides the means of predicting the rate of change under selection. A programme of selective breeding was carried out with the objects of finding out if the mean susceptibility changed at the rate predicted, and of exploring the possibility of producing strains with susceptibilities differing widely enough for the strains to be useful in other studies of lung tumours. This selection experiment is the subject of the present paper. Selection was applied both for increased and for decreased susceptibility, and in both cases the susceptibility changed at about the predicted rate. Nine generations of upward selection, and six of downward, produced strains almost as divergent in susceptibility as the most extreme of the available inbred strains, A and C57BL.

### METHODS

### *Strain*

The random bred strain to which selection was applied was the strain designated LX. This strain was constructed from crosses between four strains that had been previously selected for large body size. The foundation generation of the selection experiment consisted of the progeny of the $F_2$ of the crossing. The body weights of this strain averaged about 28 g. in females and 30 g. in males at 6 weeks of age ; the number of live young born in first litters averaged about 10.

### *Treatment*

For the induction of lung tumours urethane was administered by intraperitoneal injection of 10 per cent aqueous solution. Two injections were given, 0·1 ml. of solution at 3 weeks of age and 0·28 ml. at 9 weeks of age. The same dose was given to all mice, irrespective of weight. Mice to be used as parents were mated at 12–14 weeks of age, which allowed an interval of at least 3 weeks for the elimination of the urethane before pregnancy ensued. The mice were killed and dissected at 23 weeks of age, i.e. 14 weeks after the final injection, and the tumours visible on the surface of the lungs were counted. Further details of the technique were given by Falconer and Bloom (1962). With this treatment males and females did not differ in the average number of tumours induced, and the sex of the mice was disregarded in all the computations.

* Agricultural Research Council, Unit of Animal Genetics.

*Breeding and selection*

The procedure for selection was complicated by the fact that no offspring could be obtained from mice after their tumours had been counted. The mice had therefore to be mated, and offspring obtained, before the tumour counts on which selection was to be based were known. Thus selection had to be made " retrospectively ", the already existing litters of the mice finally selected being retained and the remainder discarded. Furthermore, it was not possible to select individual mice, and instead the best of the already mated pairs had to be selected. This inevitably resulted in some desirable mice having to be rejected because they had undesirable mates. The procedure was as follows.

The foundation generation, designated generation O, consisted of 33 litters from single-pair matings. Approximately two males and two females from each litter were treated with urethane, and tumour counts were obtained from a total of 118 mice from these litters. These counts provide the estimate of the mean susceptibility of the strain before selection. Thirty-five single-pair matings were made among, as nearly as possible, one male and one female from each of these litters when they were between 10 and 12 weeks old, i.e. shortly after the completion of their urethane treatment. The matings were made at random, but with the avoidance of brother-sister or cousin matings. These were the pairs to which the first selection was applied when their tumour counts were known. Meantime their first litters were weaned at 3 weeks, and approximately 2 males and 2 females from each litter were treated with urethane in preparation for the next generation. When the tumour counts of the parents were known, there were 32 pairs available for selection, one pair having proved sterile and 2 pairs having lost one member by death before the tumours were counted. The mean susceptibility of each pair available for selection was calculated, and the 15 pairs with the highest susceptibilities were selected. As soon as the youngest offspring of the selected pairs were 10 weeks of age, that is when an interval of at least one week had elapsed after the second urethane injection, they were mated at random in 30 pairs to provide for the next generation. These offspring of the selected pairs, when eventually their tumours had been counted, provided the estimate of the mean susceptibility of the first selected generation (generation 1), the difference from the foundation generation being the result of one generation of selection. The amount of selection applied—i.e. the selection differential—was given by the average superiority of the selected pairs ; that is to say the difference between the mean of the 15 selected pairs and the mean of the 118 mice constituting the foundation generation.

The procedure applied to the succeeding generations was basically the same as that described in detail for the first generation above. In the strain selected for high susceptibility, 15 pairs were always selected and the strain continued from their progeny. The 15 pairs were ideally selected out of 30 pairs, but some mice died before their tumours were counted and a few pairs were sterile, so that in practice there were always fewer than 30 pairs available for selection. The mean susceptibility of each generation was calculated from the tumour counts of the offspring of the 15 selected pairs. Ideally there were 60 offspring measured for susceptibility, but in practice there were fewer as a result of deaths before the tumours were counted. The generation means were calculated from all the mice measured, including those whose mates had died and those in sterile matings. The selection differential was calculated as the difference between the mean susceptibility of the 15 selected pairs and the mean of all mice measured in their generation. The numbers of mice measured in each generation and the numbers of pairs available for selection are listed in Table I. Selection for high susceptibility was carried on for 9 generations, occupying a period of 2½ years.

TABLE I.—*Numbers of Mice Measured and Mean Susceptibilities in Square Root Units*

| Strain and generation | All mice measured No. | All mice measured mean | No. of pairs available | Pairs selected No. | Pairs selected mean | Selection differential | Reversed selection: Offspring measured No. | Offspring measured mean | Pairs selected No. | Pairs selected mean | Selection differential |
|---|---|---|---|---|---|---|---|---|---|---|---|
| *High* | | | | | | | | | | | |
| 0 | 118 | 2·55 | 32 | 15 | 3·07 | +0·52 | .. | | 17 | 1·75 | −0·80 |
| 1 | 57 | 2·83 | 27 | 15 | 3·61 | +0·78 | 67 | 2·10 | .. | | .. |
| 2 | 56 | 2·80 | 25 | 15 | 3·24 | +0·44 | .. | | .. | | .. |
| 3 | 55 | 2·75 | 26 | 15 | 3·35 | +0·60 | .. | | .. | | .. |
| 4 | 60 | 3·70 | 27 | 15 | 4·11 | +0·41 | .. | | 11 | 2·99 | −0·71 |
| 5 | 57 | 3·76 | 26 | 15 | 4·53 | +0·77 | 41 | 3·31 | .. | | .. |
| 6 | 49 | 4·18 | 19 | 15 | 4·37 | +0·19 | .. | | .. | | .. |
| 7 | 53 | 4·66 | 25 | 15 | 4·96 | +0·30 | .. | | .. | | .. |
| 8 | 51 | 4·96 | 23 | 15 | 5·56 | +0·60 | .. | | .. | | .. |
| 9 | 56 | 4·99 | .. | .. | | .. | .. | | .. | | .. |
| *Low* | | | | | | | | | | | |
| 3 | 55 | 2·75 | 26 | 10 | 1·98 | −0·77 | .. | | .. | | .. |
| 4 | 37 | 2·54 | 17 | 10 | 1·91 | −0·63 | .. | | 7 | 3·01 | +0·47 |
| 5 | 36 | 2·34 | 12 | 10 | 1·90 | −0·44 | 25 | 3·48 | .. | | .. |
| 6 | 36 | 2·11 | 16 | 10 | 1·58 | −0·53 | .. | | .. | | .. |
| 7 | 38 | 1·77 | 19 | 10 | 1·18 | −0·59 | .. | | .. | | .. |
| 8 | 37 | 1·28 | 17 | 10 | 0·68 | −0·60 | .. | | .. | | .. |
| 9 | 35 | 0·83 | .. | .. | | .. | .. | | .. | | .. |
| *Control* | | | | | | | | | | | |
| 0 | 118 | 2·55 | .. | .. | | .. | .. | | .. | | .. |
| 4 | 73 | 2·55 | .. | .. | | .. | .. | | .. | | .. |
| 6 | 47 | 2·33 | .. | .. | | .. | .. | | .. | | .. |
| 7 | 53 | 2·33 | .. | .. | | .. | .. | | .. | | .. |
| 8 | 54 | 2·69 | .. | .. | | .. | .. | | .. | | .. |
| 9 | 58 | 2·55 | .. | .. | | .. | .. | | .. | | .. |
| All | 403 | 2·51 | .. | .. | | .. | .. | | .. | | .. |

On three occasions the procedure was modified so as to provide reversed selections, as follows. Normally the offspring of the pairs that had not been selected were discarded, and their tumours were not counted. In generations 0, 3 and 4, however, they were retained and their tumours were counted. Their mean susceptibility thus showed the effect on one generation of selection for low susceptibility. In the 3rd generation these offspring of the least susceptible parents were mated and a low-susceptibility strain started from them. Selection for low susceptibility was continued in this strain for 6 generations. This strain was selected by the same procedure as the high-susceptibility strain, but it was maintained by smaller numbers, 10 pairs being selected for low susceptibility out of, ideally, 20 pairs available for selection, but in practice out of fewer.

In addition to the two selected strains, a control strain was maintained without selection, but the tumour numbers were not counted in every generation. Samples of about 50 to 70 mice of the control strain were treated and their tumours counted in generations 4, 6, 7, 8 and 9. The control strain was maintained with minimal inbreeding by 20 pairs in each generation.

*Transformation to square-roots*

The tumour number of each individual was converted to its square root, and all subsequent calculations were made on the square roots of the tumour numbers. The mean of each pair, which was the criterion of selection, was the mean of the square roots, and the results of the selection are given in terms of square roots. This transformation to the square root scale was made for two reasons. First, the rate of response was expected to be greater because the heritability was higher when based on the square roots of tumour numbers than when based on the tumour numbers themselves—54·5 per cent as against 48·7 per cent (Falconer and Bloom, 1962). This means that the square root gives a better estimate of an individual's ability to transmit its higher (or lower) susceptibility to its offspring, and consequently the selection would be expected to be more effective when based on square roots. The transformation would not, of course, affect the choice of individual mice, but it might sometimes affect the choice of pairs based on the mean of the pair. The second reason for making the change of scale was that the transformation to square roots rendered the distribution nearly symmetrical. Without the transformation, the very asymmetrical distribution of tumour numbers would complicate the interpretation of the result of selection, because the heritability would be expected to change when the mean tumour number changed, as explained in our earlier paper (1962).

### RESULTS

*Response to selection*

The mean susceptibility in square root units, and the selection differentials applied, in each generation are given in Table I, and are also shown graphically in Fig. 1. The generation means are plotted in the figure against the cumulated selection differential. That is to say the mean susceptibility of any particular generation is plotted against the total amount of selection applied to all previous generations, obtained simply by adding together the previous selection differentials. The results are plotted in this way, instead of by generation number, because the selection differentials varied considerably from generation to generation, and the response is expected on theoretical grounds to be proportional to the selection differential. The mean susceptibility of the unselected control strain in the generations measured is shown by crosses in the figure placed in positions roughly corresponding to the generations of the selected strains with which they were contemporaneous, though of course no selection was applied to the control strain.

The results of the selection are quite clear and straightforward. Selection was effective in changing the mean susceptibility both up and down. Starting from a mean of 2·55 square root units, the high-susceptibility strain reached a level of 4·99 units after 9 generations of selection, and the low-susceptibility strain, starting from a level of 2·75 units was reduced to 0·82 units after 6 generations of selection. The means of the actual tumour numbers in the final generations were 26·8 tumours in the high-susceptibility strain, and 1·5 tumours in the low, while the mean of the unselected control strain was 7·0 tumours. The differences produced by the selection are shown also by the histograms in Fig. 2, which give the distributions of tumour numbers in the three strains. The last 2 generations are combined in the distribution of the high-susceptibility strain because they did not differ much in mean. The distribution in the low susceptibility strain is based on the final generation only, and that of the control on all the generations measured. It is noteworthy that there was hardly any overlap in tumour number between the mice of the two selected strains.

Fig. 1.—Responses to selection for the number of lung tumours induced by urethane. Selection for increased susceptibility is shown by solid lines, selection for decreased susceptibility by broken lines. The points refer to the means of all mice in successive generations, plotted on the vertical scale as the mean of the square roots of the number of tumours. (The right-hand vertical scale is the square of the left-hand scale, and shows approximately the mean number of tumours.) The horizontal scale shows the total amount of selection applied, up to the generation plotted. The generations are numbered beside each point. The sloping straight lines are the predicted responses based on the previously estimated heritability of 54·5 per cent.

The predicted responses to selection are shown by the sloping straight lines in Fig. 1. Details of how the prediction is made can be found in Falconer (1960). It will suffice here to say that the predicted response is given by the formula $R = h^2S$, where $R$ is the response, $h^2$ is the heritability, and $S$ is the selection differential. The lines drawn are therefore simply lines with a slope, positive or negative according to the direction of selection, equal to the heritability of 0·545 as estimated from the LX strain in the previous study (Falconer and Bloom, 1962). The observed responses show the irregularities usually found in selection experiments, but the overall agreement between the observed and predicted responses is good, particularly in the high-susceptibility strain. The low-susceptibility strain responded at almost exactly the predicted rate in the later generations but not at the beginning. The chief anomaly seems to be in the position of generation 3 as the starting point for the downward selection. The subsequent response of the high-susceptibility strain and the results of the reversed selections in generation 4 strongly suggest that the observed susceptibility of generation 3 was below the genetic value for this generation, and this would account for the apparent failure of the low-susceptibility strain to respond at the predicted rate at the beginning.

It should be mentioned that the heritability on which the predicted response is based was estimated in the previous study from the regression of offspring on their parents, and that half of these data came from the mice shown here as

FIG. 2.—Frequency distributions of the number of lung tumours in the control strain (all generations measured), the high-susceptibility strain (last two generations combined), and the low-susceptibility strain (last generation only). The figures in brackets are the numbers of mice.

parents and offspring in generations 0 and 1, the remainder coming from the 15 selected pairs of parents in generations 1 and 2 with their offspring in generations 2 and 3. The regression of offspring on parents was, however, calculated within generations so that the response to selection in these generations did not contribute to the estimate of the heritability to be used in predicting the response.

*Growth and fertility*

It is of interest to find out whether the changes of susceptibility brought about by selection had any effect on the growth or fertility of the mice. An analysis of the correlation between tumour number and weight at various ages (Bloom, in press) showed that there was a negative correlation between tumour number and 3-week weight, but no correlation with weight at other ages independently of 3-week weight. Consequently, if any part of the correlation with 3-week weight were genetically determined, it might be expected that the high-susceptibility strain would show a reduction of 3-week weight, and the low-susceptibility strain an increase, in comparison with the control, as a result of the changes of susceptibility. Adult weights might be expected to show a change correlated with the change of 3-week weight. The mean weights of the mice at 3 weeks and also at 9 weeks of age are shown in Fig. 3. It is apparent that the 3-week weights did differ in the expected direction in every generation but one, the high-susceptibility strain having the lower weights. The mean differences and their standard errors in generations 4 to 9 inclusive, were 1·55 $\pm$ 0·51 g. in females and 1·39 $\pm$ 0·53 g. in males. The mean weights at 9 weeks differed in the same direction, though less regularly. The differences at 9 weeks were probably no more than a reflection of the differences at 3 weeks.

The difference between the strains in 3-week weight, though consistent with the previously determined correlation, cannot with certainty be attributed to

FIG. 3.—Mean weights of the mice in successive generations, at 3 weeks and at 9 weeks of age.

the differences of tumour number brought about by selection, for two reasons. First, the difference between the selected strains did not become greater in the later generations when the difference of tumour number was greater, and, second, the control strain was not very clearly intermediate between the two selected strains. The weights of mice at 3 weeks of age are influenced by the number of young in the litter, and it is possible that the differences between the strains were, in part at least, the consequence of a difference of litter size.

The mean number of young born alive in first litters is shown for each generation in Fig. 4. The graph suggests that the strains did differ in average litter size, the high-susceptibility strain having the largest litters and the control the smallest. An analysis of variance of the litter sizes in generations 4 to 9 showed that the differences between the strains were significant at the 5 per cent level. It seems rather unlikely, however, that the differences were directly associated with the changes of susceptibility, because the ranking of the strains is not the same for litter size as for susceptibility and the litter sizes, like the 3-week weights, did not diverge regularly as the susceptibilities diverged. The numbers of young alive at weaning showed the same general pattern as the litter sizes at birth, the average loss between birth and weaning being about one mouse per litter.

FIG. 4.—Mean fertility of females in successive generations, measured as the number of young born alive in first litters. Sterile females are excluded.

### DISCUSSION

The first object of the experiment was to find out if the change of susceptibility produced by selective breeding agreed with the prediction based on the previous genetical analysis of the strain. The good agreement found between the observed and predicted responses serves to verify the conclusions of the previous study, but is chiefly of genetical interest in providing additional evidence of the validity of the theory of selection responses.

The discovery (Bloom and Falconer, in press) that the two strains, A and C57BL differ by a single gene with a large effect on susceptibility to urethane-induced tumours raises the question of whether any of the response to selection could be attributed to this gene, or to any other single gene with a large effect, present in the LX strain. Evidence on this question can be obtained from the distribution of tumour numbers in the LX strain shown in Fig. 2. By definition, a major gene is a gene with an effect large enough to be detectable against the background of variation caused by other genes and non-genetic factors. Therefore if a major gene is segregating the distribution will be bimodal (or trimodal if the gene is incompletely dominant). The distributions of tumour numbers show no indication of bimodality either in the unselected LX strain or in the selected strains, and there is therefore no evidence of the segregation of a major gene. The difference of susceptibility between the two strains that was built up by selection must therefore be attributed to differences at a number of gene loci, none of which have individually a large effect. The number of gene loci involved cannot, however, be determined from the present data ; nor can their individual properties be known, because none was recognizable individually either by its effect on susceptibility or by pleiotropic effects on other characters. It is possible that some of the genes causing differences of susceptibility could have originated from the mutagenic action of urethane ; but it is much more likely that they were present in the strain before the treatment with urethane was started, because if a significant amount of new genetic variability had arisen by mutation in each generation the heritability and the rate of response to selection would have increased, and they did not do so.

The second object was to explore the possibility of producing potentially useful strains by selective breeding. The question here is whether the directed effort of selection gives better results than the undirected processes of inbreeding. Genetic differences between inbred strains arise during the inbreeding by an essentially random process. Differentiation in the right direction can be aided by selection during the early stages of inbreeding, but the efficacy of the selection is severely restricted by the inbreeding, and the extent of the difference between two inbred strains chosen for any particular purpose depends mainly on the number of strains that have been screened for their suitability. Selection without inbreeding, in contrast, produces genetic divergence in the desired direction. The results of the experiment from this point of view can be assessed by comparison of the levels of susceptibility attained by selection with those of the two most extreme of the currently available inbred strains. This comparison is probably most easily appreciated if made in terms of the mean number of tumours without transformation to square roots. The mean numbers of tumours in the last generation of selection were 26·8 tumours in the high-susceptibility strain and 1·5 tumours in the low susceptibility strain. The two most divergent inbred strains, treated by the same method gave mean tumour numbers of 23·7 in the A strain and 0·91 in the C57BL strain (Falconer and Bloom, 1962). Thus selection for increased susceptibility continued over 9 generations produced a strain with a susceptibility exceeding that of the highest inbred strain, and selection for decreased susceptibility continued for 6 generations produced a strain with a susceptibility nearly as low as that of the lowest inbred strain. The experiment therefore demonstrates clearly that, as a means of producing divergent strains, selection in a genetically heterogeneous strain compares very favourably with the screening of inbred strains which have become differentiated by the random process of inbreeding.

Circumstances did not permit the continuation of the selection programme beyond the 9th generation and the experiment therefore does not reveal the full potentialities of selective breeding. The decrease of susceptibility could probably not be taken very much further in the low-susceptibility strain, unless the technique of tumour induction were modified so as to give a larger number of tumours, because with the technique used there were already about 50 per cent of animals with no tumour and there was consequently not much phenotypic variability on which the selection could operate. The increase of susceptibility in the high-susceptibility strain would, however, be expected to continue much further. The question of how far the mean susceptibility might have been increased if the programme had been continued can be partially answered by reference to other selection experiments. Most selection experiments have continued to yield progress for about 20 generations (Falconer, 1960), so the present experiment has probably realized not more than half of the increase of susceptibility than could be attained with the genetic variability present in the strain. In making comparisons with other selection experiments, however, it is necessary to take account of the intensity of selection, because with more intense selection the total possible progress will be achieved in a shorter time. In most other experiments, at least those with mice, it is usually possible to select about 25 per cent of the animals measured and reject 75 per cent, but in the present experiment rather more than 50 per cent had to be selected and less than 50 per cent rejected. The intensity of selection was therefore much less than is usually achieved, and consequently the rate of progress toward the ultimate limit must have been slower than in other experiments. It can therefore be asserted with some confidence that less than half of the total possible increase of susceptibility has been achieved, and that if the selection were continued the mean could be increased to at least 7·7 square root units, or about 60 in actual tumour numbers.

The speed of progress achieved in this experiment could have been considerably greater if the facilities available had allowed a more intense selection to be applied. The selection of 15 pairs was decided by considerations of inbreeding. Inbreeding during selection not only reduces the reproductive performance of the strain, but also limits the progress ultimately attainable by the selection. The use of 15 pairs of parents with mating for minimal inbreeding gives a rate of inbreeding of 0·83 per cent per generation, or about 30 generations to give the equivalent of one full-sib mating, which was thought to be an acceptable rate. The facilities available limited the number of animals treated to 30 pairs out of which the 15 pairs were to be selected. If it had been possible to treat and count the tumours of all the first-litter offspring of the selected pairs, about 60 pairs would have been available out of which to select 15. This would have given an intensity of selection of 25 per cent and the speed of progress would have been approximately doubled.

Lung tumours are, of course, a very favourable form of cancer for the study of selective breeding because the number of tumours gives a graded response to the carcinogen, which allows the susceptibility of individual animals to be measured. If selection were to be applied to other forms of cancer the procedure would have to be modified, particularly when only a single tumour is formed. If the incidence were fairly high, the susceptibility of individuals might still be measured as a graded response, by the age of onset, and the same procedure of selection could then be applied. But if the incidence of single tumours were low, family selection would have to be applied. Whole families are then selected or rejected on the basis of the incidence in the family and this means more space is required if the rate of inbreeding is to be kept to an acceptably low level. Even in these more difficult circumstances, however, selection should prove a more effective method of changing susceptibility than the screening of randomly inbred strains.

### SUMMARY

Selective breeding for the number of lung tumours induced by intraperitoneal injection of urethane was applied to a genetically heterogeneous strain of mice. The strain was selected for increased tumour number over nine consecutive generations, and the mean tumour number increased from 7·0 in the unselected strain to 26·8 in the 9th generation. The susceptibility of this selected strain was in excess of that of the inbred A strain, which under the same treatment had a mean tumour number of 23·7. Reasons are given for believing that if the selection had been continued a mean tumour number of at least 60 would eventually have been attained.

After three generations of selection the strain was split and selection for decreased tumour number was applied to one branch for a further six generations.

The mean tumour number in the last generation was 1·5, and the susceptibility was reduced nearly to the level of the inbred C57BL strain, which under the same treatment had a mean tumour number of 0·91.

The criterion of selection in both strains was the mean of the square roots of the tumour numbers of mated pairs of mice. The rate of response to the selection agreed well with the rate predicted from the previously estimated heritability of 54·5 per cent.

We are grateful to the Medical Research Council for the provision of a grant which enabled this work to be done, and to Miss Lucile Shiels for technical assistance.

### REFERENCES

Bloom, J. L.—*J. nat. Cancer Inst.* (in press).
*Idem* and Falconer, D. S.—*Ibid.* (in press).
Falconer, D. S.—(1960) 'Introduction to Quantitative Genetics'. Edinburgh (Oliver & Boyd).
*Idem* and Bloom, J. L.—(1961) *Nature, Lond.*, **191**, 1070.—(1962) *Brit. J. Cancer*, **16**, 665.